# 1-Diphenyl­methyl-4-ethyl­piperazine-1,4-diium dichloride

**DOI:** 10.1107/S1600536810024530

**Published:** 2010-06-30

**Authors:** Hong-Yun Qiao, Su-Hai Xu, He-Xia Jiang

**Affiliations:** a Institute of Functional Biomolecules, State Key Laboratory of Pharmaceutical Biotechnology, Nanjing University, Nanjing 210093, People’s Republic of China; b Department of General Surgery, Center of Minimally Invasive Surgery, the 81st Hospital of PLA, Nanjing 210002, People’s Republic of China

## Abstract

In the title compound, C_19_H_26_N_2_^2+^·2Cl^−^, the piperazinediium ring exhibits a chair conformation. The dihedral angle between the two benzene ring planes is 76.45 (13)°. Both amine-group H atoms participate in hydrogen bonding with the two Cl atoms.

## Related literature

The title compound was obtained in our search for a strong anti-*Helicobacter pylori* secondary metabolite. For general background to *H. pylori*, see: Gebert *et al.* (2003 ▶); Li *et al.* (2007 ▶); Moran & Upton (1986 ▶). For bond lengths and angles in related structures, see: Raves *et al.* (1992 ▶); Ilangovan *et al.* (2007 ▶).
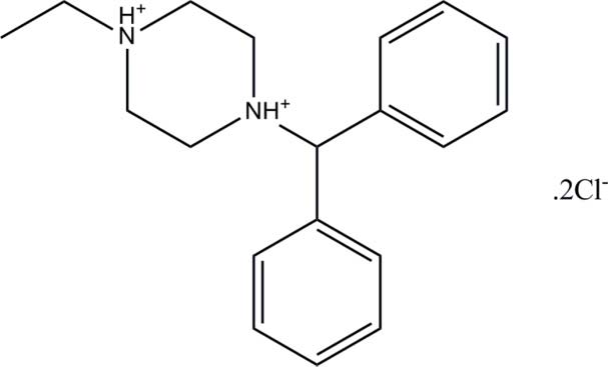


## Experimental

### 

#### Crystal data

C_19_H_26_N_2_^2+^·2Cl^−^
*M* *_r_* = 353.32
Monoclinic, 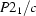

*a* = 15.069 (3) Å
*b* = 7.2950 (15) Å
*c* = 18.565 (4) Å
β = 106.35 (3)°
*V* = 1958.3 (7) Å^3^
*Z* = 4
Mo *K*α radiation
μ = 0.33 mm^−1^
*T* = 293 K
0.30 × 0.20 × 0.10 mm

#### Data collection

Enraf–Nonius CAD-4 diffractometer
Absorption correction: ψ scan (North *et al.*, 1968 ▶) *T*_min_ = 0.907, *T*_max_ = 0.968
3684 measured reflections
3542 independent reflections
2101 reflections with *I* > 2σ(*I*)
*R*_int_ = 0.028
200 standard reflections every 3 reflections  intensity decay: 1%

#### Refinement

*R*[*F* ^2^ > 2σ(*F* ^2^)] = 0.062
*wR*(*F* ^2^) = 0.157
*S* = 1.03
3542 reflections
216 parameters
H atoms treated by a mixture of independent and constrained refinement
Δρ_max_ = 0.38 e Å^−3^
Δρ_min_ = −0.25 e Å^−3^

### 

Data collection: *CAD-4 Software* (Enraf–Nonius, 1989 ▶); cell refinement: *CAD-4 Software*; data reduction: *XCAD4* (Harms & Wocadlo, 1995 ▶); program(s) used to solve structure: *SHELXS97* (Sheldrick, 2008 ▶); program(s) used to refine structure: *SHELXL97* (Sheldrick, 2008 ▶); molecular graphics: *SHELXTL* (Sheldrick, 2008 ▶); software used to prepare material for publication: *SHELXTL*.

## Supplementary Material

Crystal structure: contains datablocks global, I. DOI: 10.1107/S1600536810024530/zq2043sup1.cif

Structure factors: contains datablocks I. DOI: 10.1107/S1600536810024530/zq2043Isup2.hkl

Additional supplementary materials:  crystallographic information; 3D view; checkCIF report

## Figures and Tables

**Table 1:** Hydrogen-bond geometry (Å, °)

*D*—H⋯*A*	*D*—H	H⋯*A*	*D*⋯*A*	*D*—H⋯*A*
N1—H1*B*⋯Cl2	0.96 (4)	2.09 (4)	3.028 (3)	165 (3)
N2—H2*B*⋯Cl1	0.85 (4)	2.16 (4)	3.006 (3)	174 (3)

**Fig. 1.**
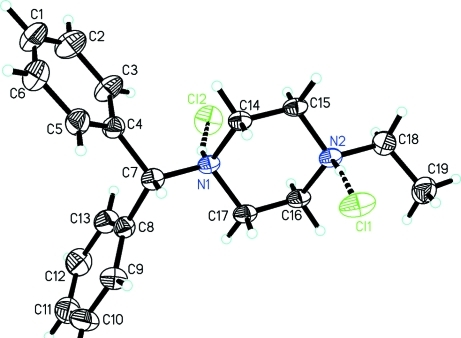
The structure of the title compound showing 30% probability displacement ellipsoids and the atom-numbering scheme.

## supplementary crystallographic information

### Comment

The human pathogenic bacterium Helicobacter pylori has been ascertained to be an antiological agent for chronic active gastritis and a significant determinant in peptic and duodenal ulcer diseases (Gebert *et al.*, 2003; Li *et al.*, 2007). Sustained infection with this bacterium could lead to development of gastric cancer (Moran & Upton, 1986). Endophytic metabolites are recognized as a versatile arsenal of antimicrobial agents, since some endophytes have been shown to possess superior biosynthetic capabilities owing to their presumable gene recombination with the host, while residing and reproducing inside the healthy plant tissues. Our particular attention was extended to anti-Helicobacter pylori constituents. A detailed bioassay-guided fractionation of the culture extract of Fusarium *sp*., an endophytic fungus in Quercus variabilis Bl., was performed to afford a strong anti-H. pylori secondary metabolite. In this paper we report the structural information for the title compound, C_19_H_26_N_2_^2+^.2Cl^-^, for which the asymmetric unit contains one 1-(diphenylmethyl)-4-ethylpiperazine-1,4-diium dication and two chloride anions. The bond lengths and angles of the title compound are in normal ranges when comparing with similar structures reported previously (Raves *et al.*, 1992; Ilangovan *et al.*, 2007). In the title compound, the piperazine fragment is in a chair conformation. The dihedral angle between the two benzene ring planes is 76.45 (13) °. Both amine-group H atoms participate in hydrogen bonding with the two Cl atoms.

### Experimental

The cultivation of Fusarium *sp*. AMB-111, an endophytic fungus in Quercus variabilis, extraction and isolation were described in a preceding communication. A residue (149 g) from the dark brown tarry mass was obtained after depositing lipids, which was then subjected to column chromatography (CC) on silica gel (1300 g, 200–300 mesh), eluting with chloroform/methanol (1:0–0:1) to give seven fractions (F-1: 28.3 g, F-2: 12.2 g, F-3: 12.5 g, F-4: 14.0 g, F-5: 13.7 g, F-6: 12.3 g and F-7: 27.4 g). F-2, showing pronounced anti-Helicobacter pylori activity, was re-chromatographed over Si-gel column eluting with chloroform/acetone (50:1–4:1) to afford four subfractions (F-2–1: 4.5 g, F-2–2: 1.4 g, F-2–3: 2.3 g and F-2–4: 2.0 g). F-2–2 was subjected to gel filtration over Sephadex LH-20 with chloroform/methanol (1:1), followed by recrystalization repeatedly to give the title compound, a yellow crystal (300 mg).

### Refinement

All H atoms were positioned geometrically (C—H = 0.93 Å for the aromatic H atoms and C—H = 0.96 Å for the aliphatic H atoms) and were refined as riding, with *U*_iso_(H) = 1.2*U*_eq_(C) and *U*_iso_(H) = 1.2*U*_eq_(N).

### Crystal data

**Table:**

C_19_H_26_N_2_^2+^·2Cl^−^	*F*(000) = 752
*M**_r_* = 353.32	*D*_x_ = 1.198 Mg m^−^^3^
Monoclinic, *P*2_1_/*c*	Mo *K*α radiation, λ = 0.71073 Å
Hall symbol: -P 2ybc	Cell parameters from 25 reflections
*a* = 15.069 (3) Å	θ = 9–12°
*b* = 7.2950 (15) Å	µ = 0.33 mm^−^^1^
*c* = 18.565 (4) Å	*T* = 293 K
β = 106.35 (3)°	Block, yellow
*V* = 1958.3 (7) Å^3^	0.30 × 0.20 × 0.10 mm
*Z* = 4	

### Data collection

**Table:**

Enraf–Nonius CAD-4 diffractometer	2101 reflections with *I* > 2σ(*I*)
Radiation source: fine-focus sealed tube	*R*_int_ = 0.028
graphite	θ_max_ = 25.3°, θ_min_ = 1.4°
ω/2θ scan	*h* = 0→18
Absorption correction: ψ scan (North *et al.*, 1968)	*k* = 0→8
*T*_min_ = 0.907, *T*_max_ = 0.968	*l* = −22→21
3684 measured reflections	200 standard reflections every 3 reflections
3542 independent reflections	intensity decay: 1%

### Refinement

**Table:**

Refinement on *F*^2^	Primary atom site location: structure-invariant direct methods
Least-squares matrix: full	Secondary atom site location: difference Fourier map
*R*[*F*^2^ > 2σ(*F*^2^)] = 0.062	Hydrogen site location: inferred from neighbouring sites
*wR*(*F*^2^) = 0.157	H atoms treated by a mixture of independent and constrained refinement
*S* = 1.03	*w* = 1/[σ^2^(*F*_o_^2^) + (0.065*P*)^2^ + 0.1129*P*] where *P* = (*F*_o_^2^ + 2*F*_c_^2^)/3
3542 reflections	(Δ/σ)_max_ < 0.001
216 parameters	Δρ_max_ = 0.38 e Å^−^^3^
0 restraints	Δρ_min_ = −0.25 e Å^−^^3^

### Special details

**Table:**

Geometry. All e.s.d.'s (except the e.s.d. in the dihedral angle between two l.s. planes) are estimated using the full covariance matrix. The cell e.s.d.'s are taken into account individually in the estimation of e.s.d.'s in distances, angles and torsion angles; correlations between e.s.d.'s in cell parameters are only used when they are defined by crystal symmetry. An approximate (isotropic) treatment of cell e.s.d.'s is used for estimating e.s.d.'s involving l.s. planes.
Refinement. Refinement of *F*^2^ against ALL reflections. The weighted *R*-factor *wR* and goodness of fit *S* are based on *F*^2^, conventional *R*-factors *R* are based on *F*, with *F* set to zero for negative *F*^2^. The threshold expression of *F*^2^ > σ(*F*^2^) is used only for calculating *R*-factors(gt) *etc*. and is not relevant to the choice of reflections for refinement. *R*-factors based on *F*^2^ are statistically about twice as large as those based on *F*, and *R*- factors based on ALL data will be even larger.

### Fractional atomic coordinates and isotropic or equivalent isotropic displacement parameters (Å^2^)

**Table:**

	*x*	*y*	*z*	*U*_iso_*/*U*_eq_	
C1	0.6307 (3)	0.4082 (10)	0.0245 (3)	0.0936 (17)	
H1A	0.6052	0.4038	−0.0273	0.112*	
C2	0.6600 (4)	0.2480 (8)	0.0637 (3)	0.0916 (16)	
H2A	0.6549	0.1369	0.0383	0.110*	
C3	0.6968 (3)	0.2534 (6)	0.1407 (2)	0.0710 (13)	
H3A	0.7158	0.1459	0.1676	0.085*	
C4	0.7053 (2)	0.4206 (5)	0.17766 (19)	0.0473 (9)	
C5	0.6766 (3)	0.5794 (6)	0.1372 (2)	0.0576 (10)	
H5A	0.6830	0.6915	0.1619	0.069*	
C6	0.6387 (3)	0.5729 (8)	0.0607 (3)	0.0816 (14)	
H6A	0.6187	0.6799	0.0338	0.098*	
C7	0.7408 (2)	0.4406 (4)	0.26239 (18)	0.0404 (8)	
H7A	0.7627	0.5672	0.2719	0.049*	
C8	0.6648 (2)	0.4179 (5)	0.30018 (19)	0.0444 (9)	
C9	0.6408 (3)	0.5678 (5)	0.3367 (2)	0.0572 (10)	
H9A	0.6747	0.6758	0.3413	0.069*	
C10	0.5665 (3)	0.5563 (7)	0.3663 (2)	0.0754 (13)	
H10A	0.5506	0.6578	0.3903	0.090*	
C11	0.5164 (3)	0.4010 (8)	0.3610 (2)	0.0766 (14)	
H11A	0.4664	0.3957	0.3810	0.092*	
C12	0.5400 (3)	0.2510 (7)	0.3258 (2)	0.0729 (13)	
H12A	0.5056	0.1437	0.3222	0.087*	
C13	0.6142 (3)	0.2571 (6)	0.2956 (2)	0.0606 (11)	
H13A	0.6300	0.1541	0.2724	0.073*	
C14	0.9003 (2)	0.3678 (4)	0.26354 (17)	0.0410 (8)	
H14A	0.9148	0.4970	0.2721	0.049*	
H14B	0.8801	0.3476	0.2097	0.049*	
C15	0.9859 (2)	0.2566 (5)	0.29714 (17)	0.0432 (9)	
H15A	0.9727	0.1281	0.2854	0.052*	
H15B	1.0342	0.2941	0.2751	0.052*	
C16	0.9435 (2)	0.2275 (5)	0.41299 (18)	0.0458 (9)	
H16A	0.9638	0.2468	0.4669	0.055*	
H16B	0.9295	0.0983	0.4039	0.055*	
C17	0.8575 (2)	0.3387 (5)	0.37957 (17)	0.0464 (9)	
H17A	0.8093	0.2996	0.4015	0.056*	
H17B	0.8705	0.4668	0.3921	0.056*	
C18	1.1074 (3)	0.1783 (5)	0.4132 (2)	0.0553 (10)	
H18A	1.1505	0.2061	0.3845	0.066*	
H18B	1.0950	0.0476	0.4091	0.066*	
C19	1.1516 (3)	0.2259 (6)	0.4946 (2)	0.0720 (13)	
H19A	1.2073	0.1561	0.5132	0.108*	
H19B	1.1095	0.1975	0.5234	0.108*	
H19C	1.1660	0.3543	0.4989	0.108*	
Cl1	1.05758 (8)	0.68359 (12)	0.40163 (5)	0.0639 (4)	
Cl2	0.80657 (8)	−0.09301 (13)	0.27873 (7)	0.0756 (4)	
H1B	0.807 (2)	0.193 (5)	0.2866 (18)	0.059 (11)*	
H2B	1.033 (2)	0.392 (5)	0.3885 (19)	0.056 (11)*	
N1	0.82357 (19)	0.3192 (4)	0.29633 (14)	0.0370 (7)	
N2	1.0189 (2)	0.2800 (4)	0.37993 (15)	0.0401 (7)	

### Atomic displacement parameters (Å^2^)

**Table:**

	*U*^11^	*U*^22^	*U*^33^	*U*^12^	*U*^13^	*U*^23^
C1	0.082 (4)	0.141 (5)	0.045 (3)	−0.003 (4)	−0.003 (2)	−0.009 (3)
C2	0.105 (4)	0.097 (4)	0.064 (3)	−0.014 (3)	0.010 (3)	−0.028 (3)
C3	0.095 (3)	0.062 (3)	0.047 (3)	−0.010 (3)	0.005 (2)	−0.012 (2)
C4	0.044 (2)	0.054 (2)	0.044 (2)	−0.0073 (19)	0.0123 (17)	−0.0052 (19)
C5	0.057 (3)	0.061 (3)	0.052 (2)	0.004 (2)	0.011 (2)	0.006 (2)
C6	0.078 (3)	0.100 (4)	0.059 (3)	0.013 (3)	0.007 (3)	0.012 (3)
C7	0.049 (2)	0.0257 (17)	0.047 (2)	−0.0090 (16)	0.0137 (17)	−0.0047 (15)
C8	0.044 (2)	0.045 (2)	0.044 (2)	−0.0045 (18)	0.0120 (17)	−0.0011 (17)
C9	0.067 (3)	0.053 (2)	0.058 (2)	−0.001 (2)	0.027 (2)	−0.0058 (19)
C10	0.081 (3)	0.085 (4)	0.071 (3)	0.010 (3)	0.039 (3)	−0.010 (3)
C11	0.062 (3)	0.108 (4)	0.064 (3)	−0.001 (3)	0.026 (2)	0.009 (3)
C12	0.060 (3)	0.086 (3)	0.076 (3)	−0.028 (3)	0.024 (2)	0.003 (3)
C13	0.064 (3)	0.062 (3)	0.057 (2)	−0.012 (2)	0.019 (2)	−0.005 (2)
C14	0.051 (2)	0.0358 (19)	0.0404 (19)	−0.0036 (17)	0.0191 (17)	−0.0005 (15)
C15	0.060 (2)	0.0354 (18)	0.0395 (19)	−0.0084 (17)	0.0220 (17)	−0.0077 (15)
C16	0.061 (2)	0.043 (2)	0.0389 (19)	−0.0094 (19)	0.0220 (18)	−0.0034 (16)
C17	0.055 (2)	0.050 (2)	0.039 (2)	−0.0078 (19)	0.0220 (17)	−0.0059 (17)
C18	0.059 (2)	0.040 (2)	0.063 (2)	0.008 (2)	0.012 (2)	−0.0043 (19)
C19	0.068 (3)	0.077 (3)	0.064 (3)	0.011 (2)	0.005 (2)	−0.001 (2)
Cl1	0.1106 (9)	0.0332 (5)	0.0560 (6)	−0.0187 (5)	0.0369 (6)	−0.0078 (4)
Cl2	0.0951 (9)	0.0287 (5)	0.0988 (9)	−0.0145 (5)	0.0205 (7)	−0.0052 (5)
N1	0.0494 (18)	0.0276 (14)	0.0358 (15)	−0.0083 (14)	0.0151 (13)	−0.0055 (12)
N2	0.0539 (19)	0.0269 (16)	0.0408 (17)	−0.0027 (14)	0.0155 (14)	−0.0052 (13)

### Geometric parameters (Å, °)

**Table:**

C1—C6	1.366 (7)	C13—H13A	0.9300
C1—C2	1.382 (7)	C14—N1	1.493 (4)
C1—H1A	0.9300	C14—C15	1.502 (4)
C2—C3	1.381 (6)	C14—H14A	0.9700
C2—H2A	0.9300	C14—H14B	0.9700
C3—C4	1.388 (5)	C15—N2	1.486 (4)
C3—H3A	0.9300	C15—H15A	0.9700
C4—C5	1.382 (5)	C15—H15B	0.9700
C4—C7	1.519 (4)	C16—N2	1.486 (4)
C5—C6	1.373 (5)	C16—C17	1.506 (5)
C5—H5A	0.9300	C16—H16A	0.9700
C6—H6A	0.9300	C16—H16B	0.9700
C7—C8	1.512 (5)	C17—N1	1.492 (4)
C7—N1	1.514 (4)	C17—H17A	0.9700
C7—H7A	0.9800	C17—H17B	0.9700
C8—C9	1.387 (5)	C18—N2	1.499 (4)
C8—C13	1.388 (5)	C18—C19	1.510 (5)
C9—C10	1.382 (5)	C18—H18A	0.9700
C9—H9A	0.9300	C18—H18B	0.9700
C10—C11	1.350 (6)	C19—H19A	0.9600
C10—H10A	0.9300	C19—H19B	0.9600
C11—C12	1.371 (6)	C19—H19C	0.9600
C11—H11A	0.9300	N1—H1B	0.96 (4)
C12—C13	1.386 (5)	N2—H2B	0.85 (4)
C12—H12A	0.9300		
			
C6—C1—C2	120.9 (4)	N1—C14—H14B	109.2
C6—C1—H1A	119.6	C15—C14—H14B	109.2
C2—C1—H1A	119.6	H14A—C14—H14B	107.9
C3—C2—C1	119.8 (5)	N2—C15—C14	111.4 (3)
C3—C2—H2A	120.1	N2—C15—H15A	109.3
C1—C2—H2A	120.1	C14—C15—H15A	109.3
C2—C3—C4	119.4 (4)	N2—C15—H15B	109.3
C2—C3—H3A	120.3	C14—C15—H15B	109.3
C4—C3—H3A	120.3	H15A—C15—H15B	108.0
C5—C4—C3	119.8 (3)	N2—C16—C17	111.1 (3)
C5—C4—C7	116.6 (3)	N2—C16—H16A	109.4
C3—C4—C7	123.5 (3)	C17—C16—H16A	109.4
C6—C5—C4	120.6 (4)	N2—C16—H16B	109.4
C6—C5—H5A	119.7	C17—C16—H16B	109.4
C4—C5—H5A	119.7	H16A—C16—H16B	108.0
C1—C6—C5	119.5 (5)	N1—C17—C16	112.3 (3)
C1—C6—H6A	120.2	N1—C17—H17A	109.1
C5—C6—H6A	120.2	C16—C17—H17A	109.1
C8—C7—N1	112.7 (3)	N1—C17—H17B	109.1
C8—C7—C4	112.2 (3)	C16—C17—H17B	109.1
N1—C7—C4	112.6 (3)	H17A—C17—H17B	107.9
C8—C7—H7A	106.3	N2—C18—C19	112.9 (3)
N1—C7—H7A	106.3	N2—C18—H18A	109.0
C4—C7—H7A	106.3	C19—C18—H18A	109.0
C9—C8—C13	118.8 (3)	N2—C18—H18B	109.0
C9—C8—C7	118.4 (3)	C19—C18—H18B	109.0
C13—C8—C7	122.6 (3)	H18A—C18—H18B	107.8
C10—C9—C8	119.9 (4)	C18—C19—H19A	109.5
C10—C9—H9A	120.1	C18—C19—H19B	109.5
C8—C9—H9A	120.1	H19A—C19—H19B	109.5
C11—C10—C9	121.4 (4)	C18—C19—H19C	109.5
C11—C10—H10A	119.3	H19A—C19—H19C	109.5
C9—C10—H10A	119.3	H19B—C19—H19C	109.5
C10—C11—C12	119.4 (4)	C17—N1—C14	108.7 (3)
C10—C11—H11A	120.3	C17—N1—C7	112.2 (3)
C12—C11—H11A	120.3	C14—N1—C7	109.4 (2)
C11—C12—C13	120.9 (4)	C17—N1—H1B	106 (2)
C11—C12—H12A	119.5	C14—N1—H1B	110 (2)
C13—C12—H12A	119.5	C7—N1—H1B	110 (2)
C12—C13—C8	119.6 (4)	C16—N2—C15	109.0 (3)
C12—C13—H13A	120.2	C16—N2—C18	113.4 (3)
C8—C13—H13A	120.2	C15—N2—C18	111.7 (3)
N1—C14—C15	112.0 (3)	C16—N2—H2B	111 (2)
N1—C14—H14A	109.2	C15—N2—H2B	107 (2)
C15—C14—H14A	109.2	C18—N2—H2B	104 (2)
			
C6—C1—C2—C3	0.9 (8)	C10—C11—C12—C13	−0.1 (7)
C1—C2—C3—C4	−1.0 (8)	C11—C12—C13—C8	−0.8 (6)
C2—C3—C4—C5	0.2 (6)	C9—C8—C13—C12	1.5 (6)
C2—C3—C4—C7	177.2 (4)	C7—C8—C13—C12	−174.2 (3)
C3—C4—C5—C6	0.8 (6)	N1—C14—C15—N2	−57.9 (4)
C7—C4—C5—C6	−176.4 (4)	N2—C16—C17—N1	57.7 (4)
C2—C1—C6—C5	0.1 (8)	C16—C17—N1—C14	−55.1 (4)
C4—C5—C6—C1	−0.9 (7)	C16—C17—N1—C7	−176.3 (3)
C5—C4—C7—C8	90.9 (4)	C15—C14—N1—C17	55.1 (3)
C3—C4—C7—C8	−86.2 (4)	C15—C14—N1—C7	177.9 (2)
C5—C4—C7—N1	−140.7 (3)	C8—C7—N1—C17	−51.4 (3)
C3—C4—C7—N1	42.2 (5)	C4—C7—N1—C17	−179.6 (3)
N1—C7—C8—C9	117.2 (4)	C8—C7—N1—C14	−172.2 (3)
C4—C7—C8—C9	−114.5 (4)	C4—C7—N1—C14	59.7 (3)
N1—C7—C8—C13	−67.0 (4)	C17—C16—N2—C15	−57.0 (4)
C4—C7—C8—C13	61.3 (4)	C17—C16—N2—C18	177.9 (3)
C13—C8—C9—C10	−1.4 (6)	C14—C15—N2—C16	57.4 (3)
C7—C8—C9—C10	174.5 (3)	C14—C15—N2—C18	−176.6 (3)
C8—C9—C10—C11	0.5 (7)	C19—C18—N2—C16	−66.6 (4)
C9—C10—C11—C12	0.3 (7)	C19—C18—N2—C15	169.8 (3)

### Hydrogen-bond geometry (Å, °)

**Table:**

*D*—H···*A*	*D*—H	H···*A*	*D*···*A*	*D*—H···*A*
N1—H1B···Cl2	0.96 (4)	2.09 (4)	3.028 (3)	165 (3)
N2—H2B···Cl1	0.85 (4)	2.16 (4)	3.006 (3)	174 (3)
